# The ISN Neuroscience School, Ondo 2024, Gene–environment interactions in neurological disorders: a contemporary update

**DOI:** 10.1242/bio.061998

**Published:** 2025-05-27

**Authors:** Victor E. Anadu, Hidaayah O. Jimoh-Abdulghaffaar, Taidinda T. Gilbert, Oluoma V. Aneke, Leviticus O. Arietarhire, Patrick C. Ichipi-Ifukor, Abiodun M. Abdullahi, Leonard S. Okah, Adeline Fanta Y. Sabine, Blessing U. Oritsetimeyin, Toheeb O. Oyerinde, Fatimo A. Sulaimon, Tolulope J. Gbayisomore, Tobiloba S. Olajide, David A. Oyeniran, Omolabake I. Omotosho, R. Oria, Olayemi K. Ijomone

**Affiliations:** ^1^ Laboratory for Experimental and Translational Neurobiology, University of Medical Sciences, Ondo, Nigeria; ^2^ Department of Physiology, Faculty of Basic Medical Sciences, College of Health Sciences, University of Ilorin, Ilorin, Nigeria; ^3^ Neuroscience Unit, Department of Veterinary Anatomy, University of Ibadan, Ibadan, Nigeria; ^4^ Babcock University, Ilishan-Remo, Ogun State, Nigeria; ^5^ Department of Biochemistry, Faculty of Science, Delta State University, Abraka, Nigeria; ^6^ Department of Human Anatomy, School of Medicine and Pharmacy, College of Medicine and Health Sciences, University of Rwanda; ^7^ Department of Human Anatomy, Faculty of Basic Medical Sciences, University of Ibadan, Nigeria; ^8^ Institute of Medical Research and Medicinal Plants Studies, Yaoundé, Cameroon; ^9^ Biomedical Science Research and Training Centre, Damaturu, Yobe, Nigeria; ^10^ Department of Anatomy, Faculty of Basic Medical Sciences, College of Health Sciences, University of Ilorin, Ilorin, Nigeria; ^11^ Department of Human Anatomy, University of Cross River State, Nigeria

**Keywords:** Environment, Genetics, Neurochemistry, Neurological disorders, Neuroscience school

## Abstract

Short summer courses focusing on the nervous system, commonly called Neuroscience Schools, are great venues for advancing training globally, particularly in low-middle income countries (LMICs). The ISN Neuroscience School attracted graduate students, postdoctoral fellows, early career researchers, and seasoned neuroscientists. The school was sponsored by the International Society for Neurochemistry (ISN) with additional support from the Company of Biologists. The school was held between 11th and 16th August, 2024, at the University of Medical Science, Ondo, Nigeria. Leading authorities in the field gave a series of talks during the event, summarizing the most recent findings on how environmental variables and genetic predispositions interact to affect the development and course of neurological illnesses. Through hands-on activities during practical sessions, participants gained a deeper knowledge of the approaches used to examine these interactions. Student pitches also promoted multidisciplinary cooperation and critical thinking by showcasing creative concepts and research ideas. The study of gene–environment interactions has several implications for the diagnosis, treatment, and prevention of neurological disorders. In this meeting report, we summarize and discuss the relevance of the school's activities while also highlighting prospects in this field in the African region.

## Teaser

**Summary:** This Meeting Review outlines emerging insights into gene–environment interactions that are reshaping our understanding of neurological disorders and opening new avenues for diagnosis, prevention, and therapy.

## Introduction

The International Society for Neurochemistry (ISN) is a society that promotes and supports the advancement of neurochemistry research and education globally. It focuses mainly on building capacity in molecular and cellular neuroscience research by exposing students and faculty members to recent and groundbreaking advances in the field. Over the years, this society has sponsored numerous scientific meetings such as this and has assembled academics (faculties and students) of various academic stages and disciplines from around the globe to partake in scientific lectures and discussions on relevant neurochemical research topics. This year's ISN School was hosted at the University of Medical Sciences, Ondo State, Nigeria, from 11–16 August 2024. The school also received additional support from the Company of Biologists.

**Fig. 1.**
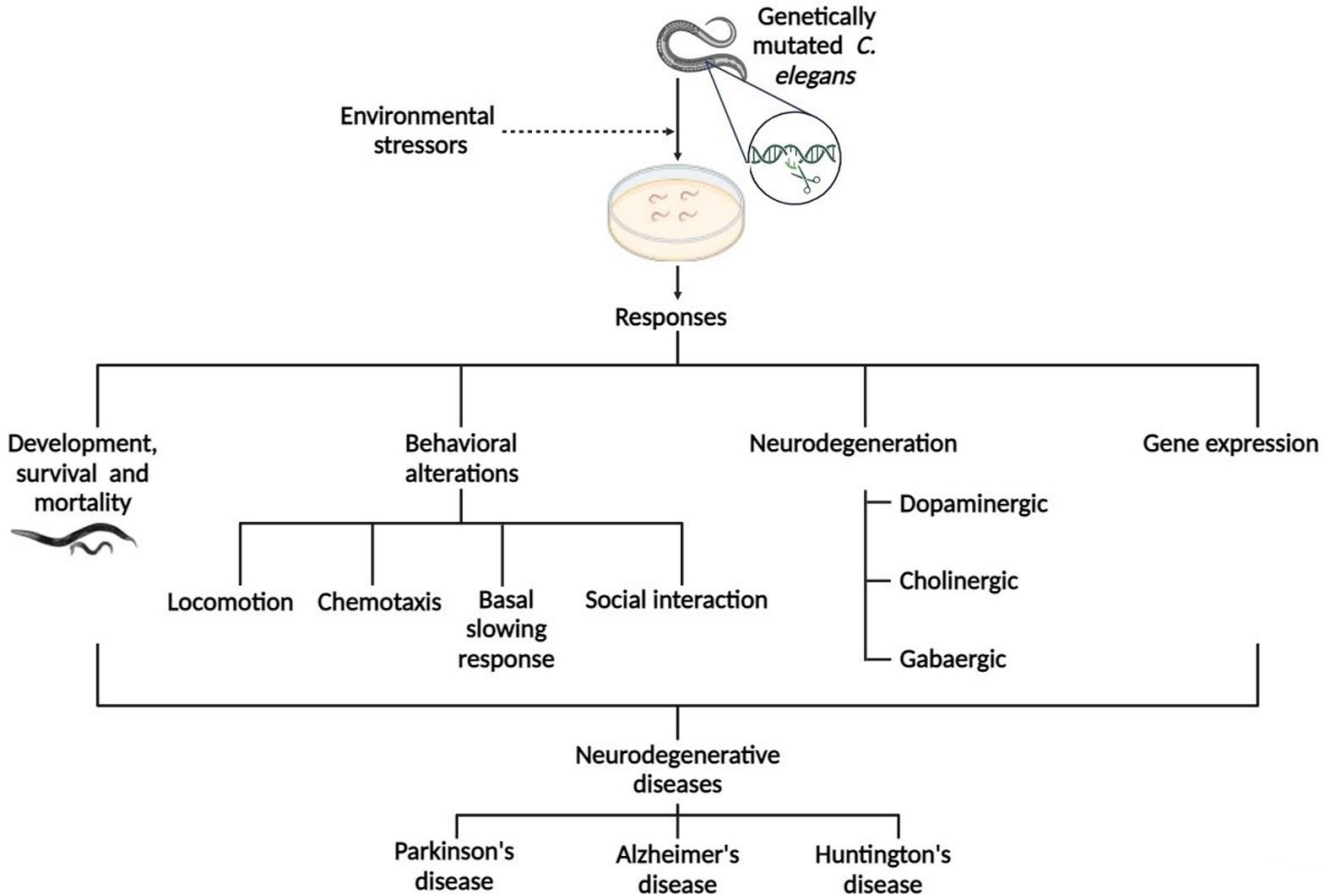
The *Caenorhabditis elegans* as a model for neurodegenerative diseases.

This school was aimed at providing a robust scholarly contemporary update on the interplay between genetic and environmental factors in the pathogenesis of various neurological disorders. Recent studies have been focused on unravelling how this complex interaction can provide relevant biological insights into the aetiology, progression, and outcomes of prevalent neurological disorders including Parkinson's disease (PD), Huntington's disease (HD), and Alzheimer's disease (AD) (Dunn et al., 2019; Wexler, 2004; Migliore and Coppedè, 2022). Prolonged and cumulated interactions between environmental and genetic risk factors have been shown to significantly influence disease risk, particularly in age-related neurodegenerative diseases (Pang et al., 2019). However, the intricacies involved in these interactions are yet to be fully understood although some studies have suggested that this phenomenon is associated with the impairment of cellular pathways and perturbation of neuronal processes, which consequently leads to neuronal death (Pang et al., 2019; Eid et al., 2019; Machiela and Southwell, 2020). Hence, getting a solid grasp of the mechanisms implicated in the aetiology of these neurological disorders could contribute to developing relevant interventions and personalized therapeutics. Among various experimental research models, the *Caenorhabditis elegans* (*C. elegans*), has particularly been useful in modelling various human neurological diseases. This model has been employed in studying the cellular and molecular mechanisms underlying the pathogenesis of some neurodegenerative disorders (Fig. 1). The *C. elegans* has been leveraged to study relevant topics in molecular biology (Haroon et al., 2018), toxicology (Gubert et al., 2018; Ijomone et al., 2024), neuroscience (Denzel et al., 2019) and genetics (Gao et al., 2018). Its advantage over other model organisms includes its short life span and simple morphology and over 70% homology to human genes, as well as a completely mapped neural network. In addition, it is easy to grow and maintain and can be genetically manipulated without stress. The *Drosophila melanogaster* (*D. melanogaster*) is another model organism that has proven valuable in studying various neurodegenerative diseases (Huang et al., 2020; Aryal and Lee, 2019). About 75% of all human disease genes have *Drosophila* homologues (Bilen and Bonini, 2005), hence its applicability in studying neurodegenerative diseases like AD and PD.

In this Meeting Review, we provide an overview of the lectures taken by international and national faculties, interactive forums, as well as practical sessions. The hands-on practical sessions incorporated both basic and advanced research techniques involving the use of the rodent and *C. elegans* models. These sessions were aimed at equipping participants with valuable knowledge and skillsets needed to improve their research output in various areas relevant to neuroscience.

## Sociodemographic characteristics of participants

**Fig. 2.**
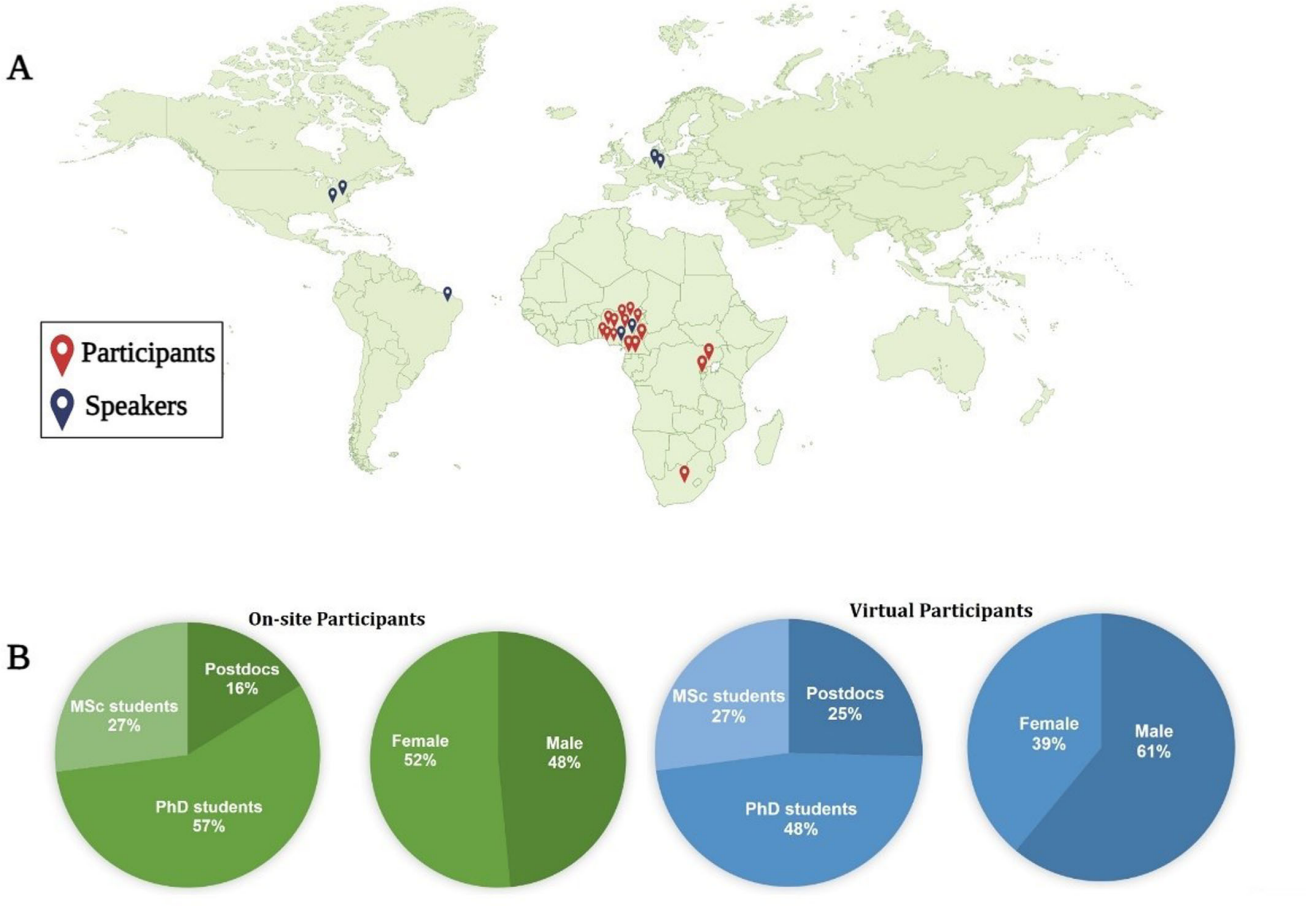
**Participant demographics of the ISN 2024 neuroscience school.** (A) Cohort of participants attended from different geopolitical regions in Nigeria and across Africa. Speakers came from across the globe (B) Gender and career stage distribution of in-person and virtual attendees.

The ISN School Neuroscience School brought together a diverse cohort of participants from different geopolitical regions in Nigeria and across Africa. The meeting adopted a hybrid format with some participants joining in person while others joined virtually. In-person attendees comprised of 33 students, 29 of whom were Nigerians from the six geopolitical zones of the country, and five participants attending from other African countries including Cameroun, South Africa, Rwanda, and Uganda. The in-person attendance had an even gender distribution comprising 16 male and 17 female participants across different academic stages including early career researchers, postdoctoral fellows, PhD, and MSc students (Fig. 2) from diverse academic disciplines such as biochemistry, anatomy, physiology, medicine, and pharmacology. Having participants from various academic disciplines enhanced the overall learning experience, leading to insightful interactive sessions and discussions as students were able to provide unique insights and perspectives from diverse research fields. In addition, 59 participants (36 males and 23 females) were selected to participate virtually. Participants in this category included graduate students (MSc and PhD), postdoctoral fellows and early career researchers (Fig. 2).

## Lectures

**Fig. 3.**
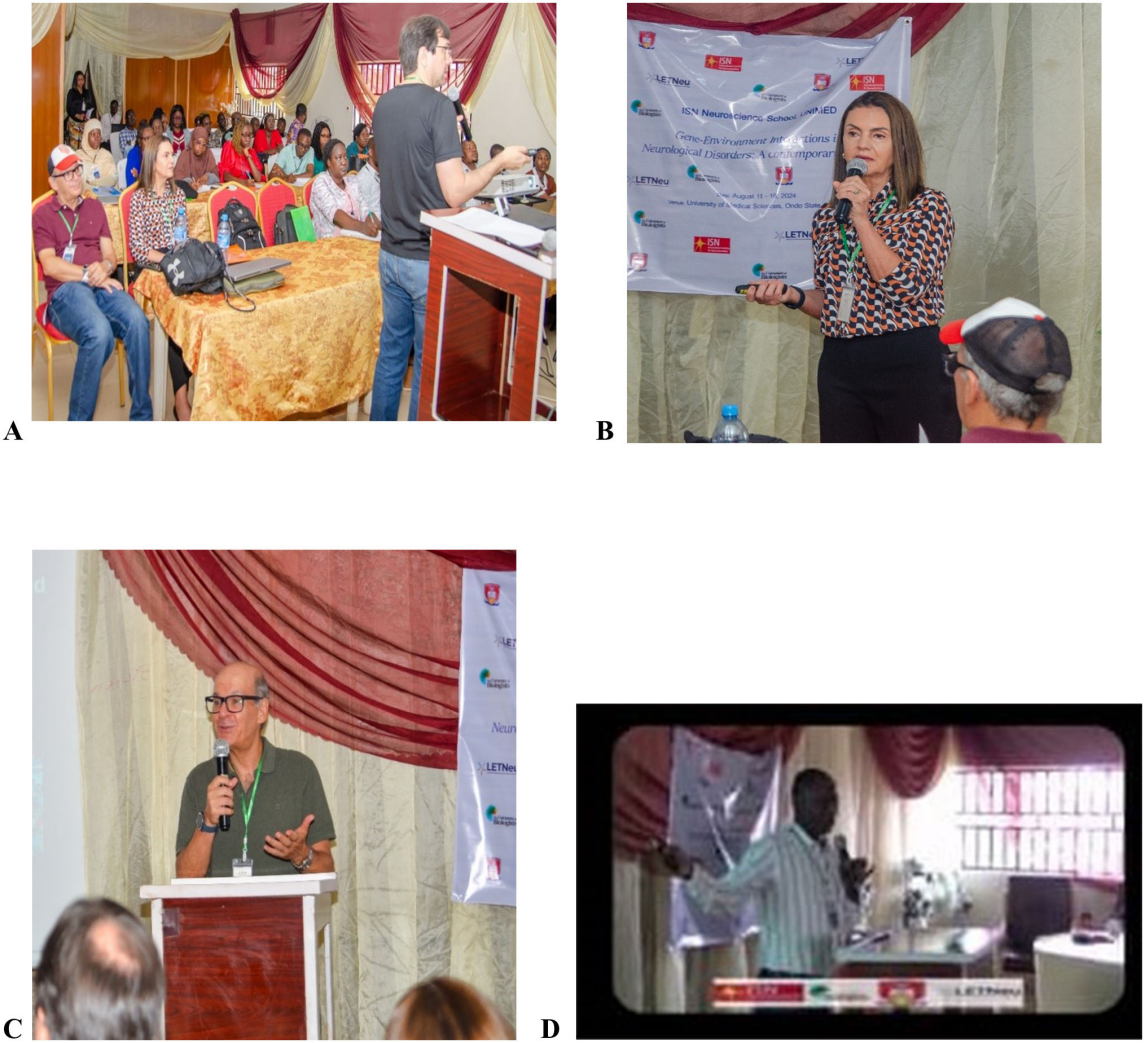
Physical lecture sessions by (A) Prof Felix Soares (B) Prof Monica Paoliello (C) Prof Michael Aschner (D) Prof Amos Abolaji.

A major highlight of the ISN Neuroscience School was the series of lectures delivered by distinguished international and local speakers attending physically and virtually. Most of the lectures evolved around the use of the *C. elegans* worm model for studying interactions between environmental and genetic components and also its applicability in neuroscience, neuropharmacology, toxicology and genetic research (Fig. 3). However, later during the school, a fresh perspective was introduced by Professors Amos Abolaji and Monica Paoliello. Professor Amos walked the participants through the use of *D. melanogaster* for studies on neurodegenerative diseases. Professor Monica Paoliello discussed major concepts and correct practices involved in reviewing literature for research.

The keynote lecture was delivered by Professor Michael Aschner, a renowned researcher from the Albert Einstein College of Medicine, USA, discussing some novel approaches to unravelling simultaneous contributions of genes and environment in brain diseases. He commenced by briefly overviewing the *C. elegans* animal model, laying emphasis on its advantages as an experimental model for research in diverse fields not limited to toxicology, pharmacology, neuroscience, and genetics. His lecture focused on elucidating mechanism through which manganese (Mn) enters the brain which is mostly via the Divalent Metal Tansporter-1 (DMT-1), resulting to neurotoxicity and pathogenesis of some neurological disorders like PD; *smf-1* and *smf-3* are relevant homologues for human DMT-1 in *C. elegans*. In his second lecture, he focused on the role of metal transporters in neurological disorders. He discussed some implications of heavy metal exposure and accumulation, as well as the interactions between metals like iron (Fe), Mn and their transporters in the brain while connecting it to the aetiology of restless leg syndrome (RLS) to metal imbalance. He also identified a gene called BTBD9, which helps regulate Fe levels in the brain. He concluded his lecture by showing that loss of function in this gene is linked with greater susceptibility to Mn-induced oxidative stress, mitochondrial dysfunction, and decreased dopamine levels (Gunter et al., 2013; Chen et al., 2022).

**Fig. 4.**
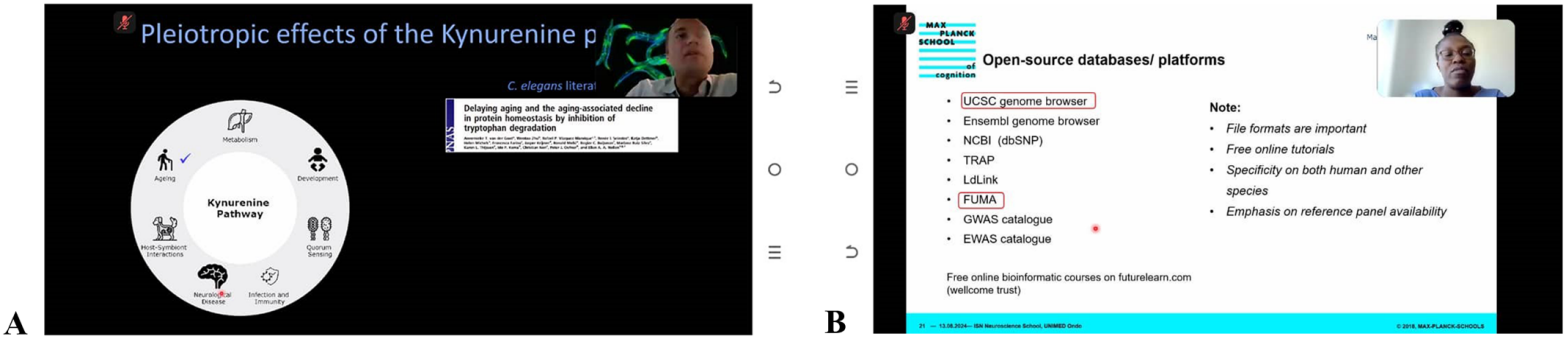
Online lecture sessions (A) Dr Alexander Benedetto (B) Mrs Comfort Ofure.

Professor Dr Julia Bornhorst from the University of Wuppertal, Germany also gave a lecture focusing on the interplay between Mn neurotoxicity and *parkin*/*PARK2* and *PINK* mutations in PD. She discussed how heavy exposure to Mn contributes to the pathogenesis and progression of PD following mutations in *parkin* and *PINK1* genes in the *C. elegans.* Her lecture highlighted the influence of these interactions on various parameters employed to study neurodegeneration in the *C. elegans* including dopamine functioning, mitochondrial dysfunction, and neuronal damage (Bornhorst et al., 2014). In addition, Mrs Comfort Ofure Okoh, a doctoral candidate with the Max Planck School of Cognition, Germany, delivered a short virtual demonstration focusing on the identification of risk variants through the use of enhancer screening tools, and how they contribute to neuropsychiatric disorders such as schizophrenia, psychosis, and epilepsy (Fig. 4b).

Professor Monica Paoliello from the Albert Einstein College of Medicine, USA, gave her lecture titled ‘Cadmium (Cd) exposure and hypertension: the role of the nervous system’. She highlighted some common sources of Cd exposure and also discussed toxicity that arises from its accumulation over time in a variety of organ systems, including the liver, kidneys, and central and peripheral nervous systems. These adverse health effects include hypertension, cardiovascular mortality, peripheral arterial disease, cancer mortality, and chronic kidney disease. She described several mechanisms that may explain the relationship between Cd exposure and altered health parameters including blood pressure, blood–brain barrier integrity, and renal functioning. Some of which were a result of predisposing factors like gender susceptibility and lifestyle choices.

Professor Félix Antunes Soares from the Federal University of Santa Maria, Brazil, delivered his lecture on modelling gene-toxicant contribution to HD using the *C. elegans* model. He started his lecture by highlighting reasons why we should leverage on the *C. elegans* animal model for neuroscience research especially for neurodegenerative diseases like HD. He highlighted some *C. elegans* strains that are used to study HD, many of which are available at the Caenorhabditis Genetics Center (CGC). He discussed extensively the role Diphenyl diselenide plays in protecting the *C. elegans* model of HD. This involves the activation of the antioxidant pathway and a decrease in protein aggregation. In addition, he showed that another substance called rutin could protect against HD through the insulin signalling pathway and autophagy activity. He concluded by highlighting future directions involving investigating the trans-generational and epigenetic implications of long-term exposure to pesticides and heavy metals in neurodegenerative diseases (Soares et al., 2017; Bicca Obetine Baptista et al., 2020; Cordeiro et al., 2020).

Dr Omamuyovwi Ijomone from the University of Medical Sciences, Ondo, discussed the impacts of metal toxicities and NRXN/NLGN mutations on neuronal perturbations in autism. His lecture commenced by identifying the burden posed by crude oil spillage and heavy metal contamination in the environment, especially in some parts of Nigeria. He further discussed various research that has studied the toxic impact of Nickel (Ni) in the rodent and *C. elegans* models. For instance, early-life exposure to Ni was shown to induce developmental and behavioural deficits in wild-type young adult *C. elegans* (Ijomone et al., 2020). The mechanism was shown to involve oxidative stress via SKN-1 (worm homologue for human Nrf2) regulation. He further described the role of Mn and Fe in modulating toxicities and neural perturbations in nrx-1/nlg-1 loss-of-function in *C. elegans* (unpublished data). In addition, he demonstrated neurological deficits that could arise as a result of co-exposure of heavy metals and stressful events in rodents' brains (Okeowo et al., 2024).

Dr Alexander Benedetto from Lancaster University delivered two lectures entitled ‘Gut-microbiome modulation of genetic risk factors in neurodegenerative diseases’ and ‘Gene–environment interplay in brain senescence’. The first lecture focused on the gut-brain axis and kynurenine pathway. In this lecture, he laid emphasis on the importance of studying this pathway, which is mostly because of the diversity of metabolites it produces. His second lecture provided insights on the evolution of neurodegenerative diseases, typically in relation to ageing. He identified the changes that take place in the brain as it ages, shedding light on the fate of key components like the synapses. In addition, he discussed various techniques applicable to the *C. elegans* model that have been developed to study brain functions (Fig. 4a). Last but not the least, Professor Amos Abolaji from the University of Ibadan, Nigeria, delivered his lecture titled ‘Assessing natural products on toxicants models of Alzheimer's and Parkinson's disease in *D. melanogaster*’. In this lecture, he discussed the suitability of the *D. melanogaster* for neurodegenerative disease studies, and why employing this model organism is critical for AD and PD research. He also discussed the roles natural products can play in rescuing chemically induced perturbations observed in these diseases. His lecture also revealed some natural products like Resveratrol that could attenuate toxicity induced in *D. melanogaster*.

## Practical sessions

We had 2 days of intensive practical sessions during the neuroscience school. The practical sessions were on *C. elegans*, rat brain dissection, rodent behavioural demonstrations and immunohistochemistry. All attendees were divided into two groups for effective learning and participation.

**Fig. 5.**
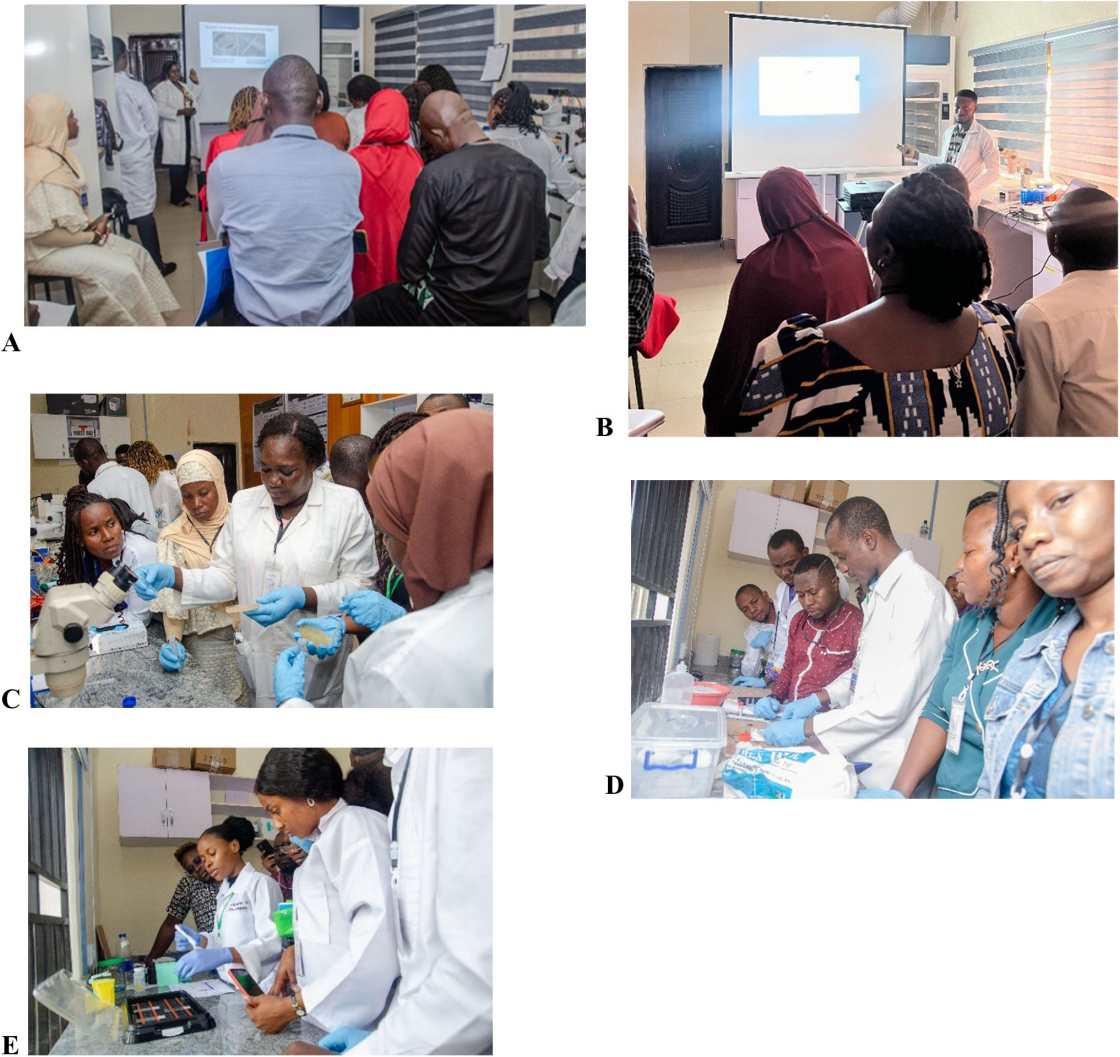
**Practical sessions.** (A) Behavioural assays (B,C) *C. elegans* (D) rodent brain dissection (E) immunohistochemistry.

The participants were taught how to properly dissect the rat brain, and how to carry out a complete immunohistochemical procedure, after which they stained a hippocampal brain region using GFAP and TNF alpha as biomarkers. Various rodent behavioural assays were also introduced to the participants. These include the open field test (OFT), novel object recognition test (NORT), Y-maze, elevated zero maze (EZM), T-maze, elevated plus maze (EPM), forepaw grip, and forced swim tests. Furthermore, the participants were briefly introduced to the *C. elegans* experimental workflow and the various assays that could be performed using this model after which they attempted the worm synchronization procedure. In all, the practical session not only offered a good learning experience, but also provided new ideas and insights for the participants (Fig. 5).

## Interactive sessions

**Fig. 6.**
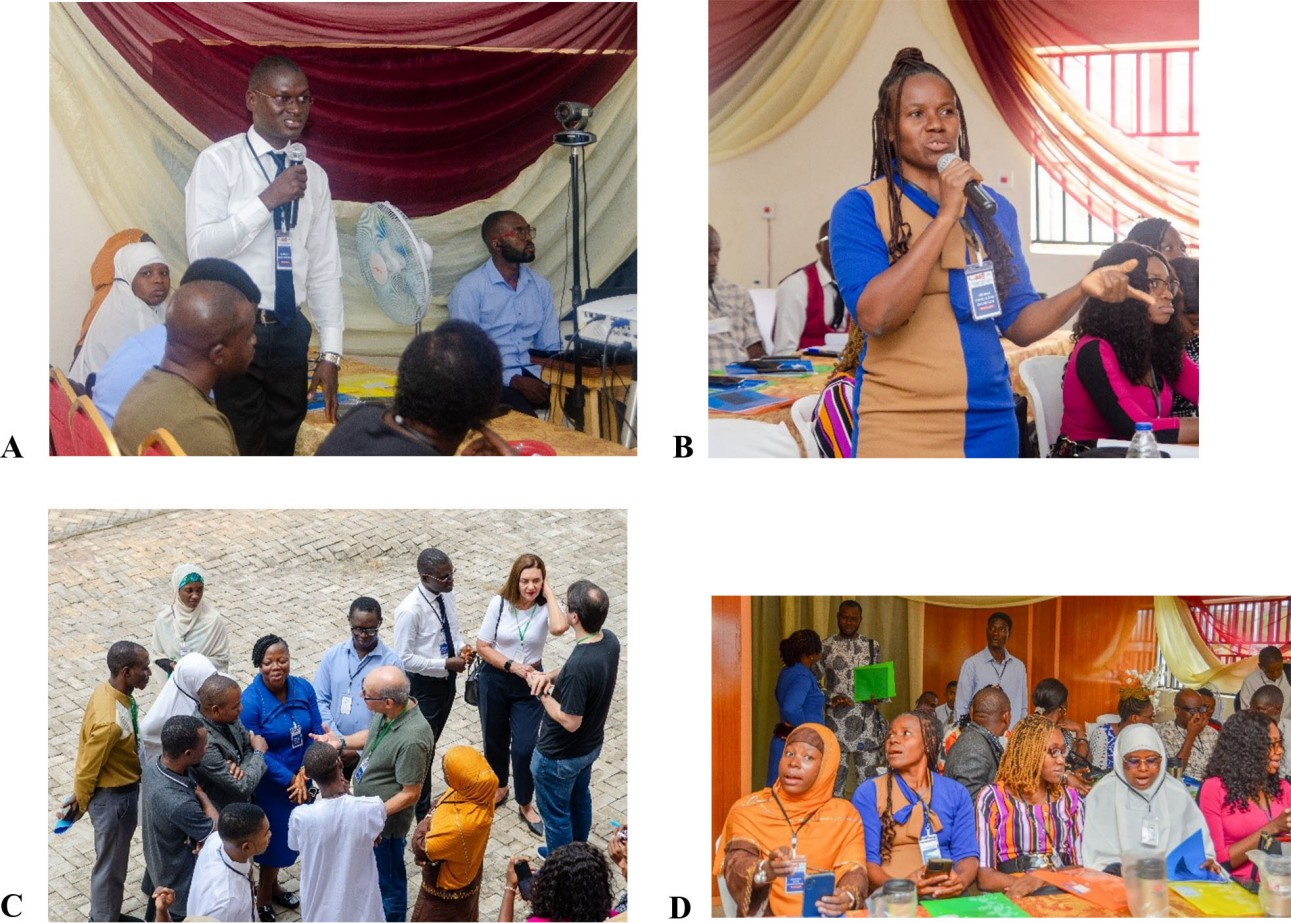
A cross-section of participants at the interactive sessions.

This session was led by Professor Amos Abolaji, Dr Kingsley Iteire, Professor Felix Soares, and Dr Omamuyovwi Ijomone, and served as an avenue for faculties-to-students and student-to-student interaction and networking. Grantsmanship was discussed during this session, alongside the various challenges faced by scientists in developing countries, and how to overcome them. Ethical issues in biomedical research were also discussed (Fig. 6).

## Tour visit

**Fig. 7.**
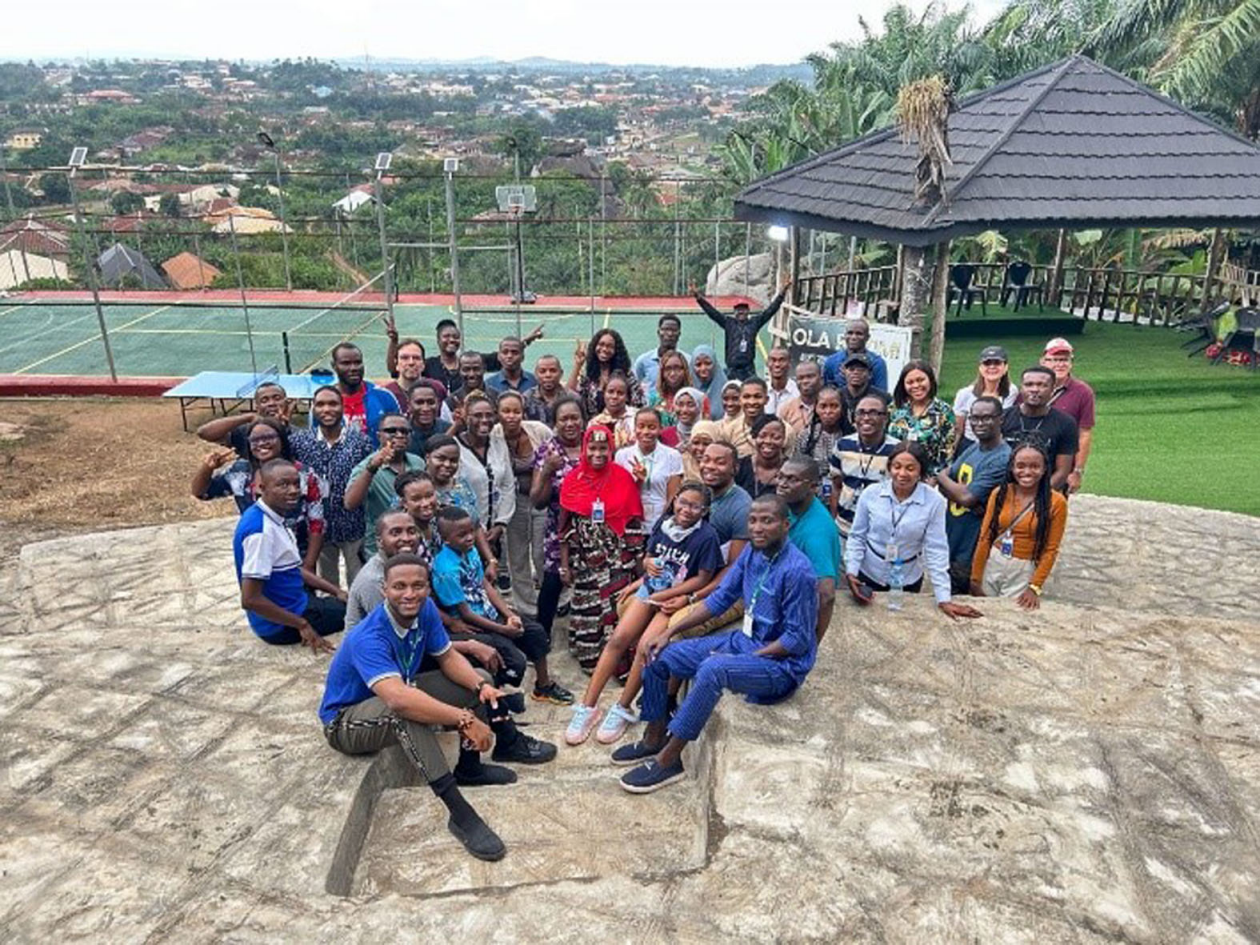
Participants and faculties at La Campagne Tropicana Resort, Ondo.

On 15 August 2024, there was a guided tour visit to La Campagne Tropicana, a stunning retreat destination situated in Ondo town (Fig. 7). This unique destination was designed with the sole purpose of showcasing and preserving African heritage, offering a deep connection to our cultural roots. The visit provided a much-needed break for the participants and faculty of the school. The hilltop location of La Campagne Tropicana not only offered a refreshing atmosphere but also gave us breathtaking panoramic views of Ondo town and its surrounding landscapes. The serene environment, combined with the rich cultural aesthetics created the perfect backdrop for relaxation and reflection. During our time there, the participants and faculty members engaged in various recreational activities, fostering friendship and relaxation. Games like table tennis, snooker and ‘shoot the basket’ were enthusiastically played, offering a fun and lively way to unwind after academic sessions.

**Fig. 8.**
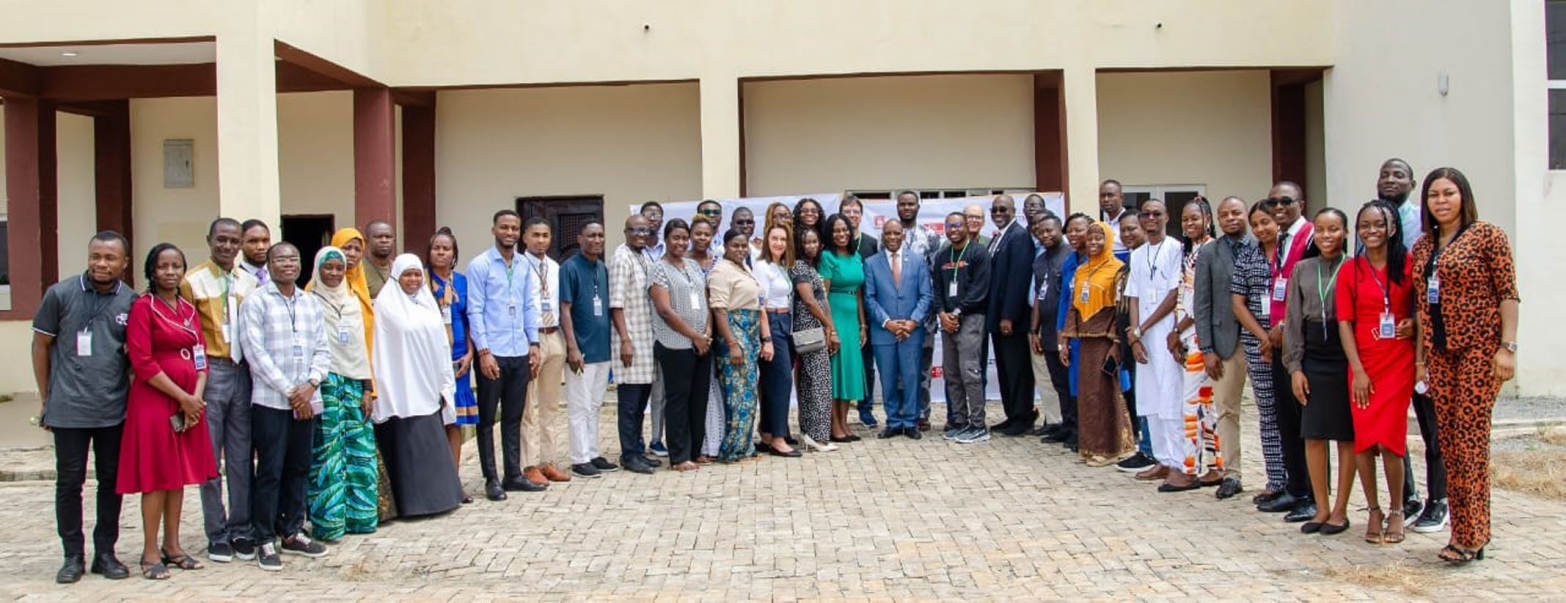
Attendees at the ISN Neuroscience School, Ondo 2024 with the management of the University of Medical Sciences (UNIMED), Ondo.

## Discussion

The ISN Neuroscience School 2024 provided a contemporary update on gene–environment interactions in neurological disorders, a field that is increasingly relevant given the rising prevalence of neurodegenerative diseases. The interaction between genetic predispositions and environmental exposures, particularly in neurodegenerative diseases like PD, AD and HD, was a recurring theme throughout the event. As underscored by keynote speaker Professor Michael Aschner, understanding how genes and environmental toxicants, such as metals like Mn and Fe, contribute to neurological disorders is crucial. His discussion on the roles of metal transporters and gene mutations, like BTBD9 in RLS, highlighted the complexity of these interactions. Professor Julia Bornhorst's investigation of parkin and PINK1 mutations in PD further emphasized the molecular mechanisms through which environmental toxins exacerbate genetic vulnerabilities, leading to neuronal dysfunction. This knowledge is critical in addressing the clinical challenges posed by neurodegenerative diseases, which are multifactorial in origin. Identifying risk factors and understanding how they synergize to drive disease progression can lead to more effective interventions. Professor Felix A. Antunes Soares's research on protective compounds like diphenyl diselenide and rutin in *C. elegans* models of HD showcases how experimental models can help elucidate potential therapeutic targets. The *Drosophila* model, presented by Professor Amos Abolaji, also demonstrated its utility in investigating neurotoxic responses and testing neuroprotective natural compounds, Professor Abolaji also emphasized the possibility of looking at sexual dimorphism in the fruit fly.

Another key theme was the impact of environmental pollutants like Cd on neurological health. Professor Monica Paoliello's discussion on the link between Cd exposure and hypertension, mediated through nervous system dysfunction, emphasized the broader health impacts of environmental toxins beyond neurodegeneration. Her insights into blood–brain barrier integrity and renal functions highlighted the importance of considering systemic health outcomes when assessing environmental toxicants' effects on the brain. Metal toxicity, particularly Mn and Ni, was a central focus in discussions on neurotoxicity. Dr Ijomone's research on early life exposure to Ni and its developmental effects in *C. elegans*, along with his work on Mn and Fe interactions in *nrx-1/nlg-1* mutations, pointed to a growing body of evidence linking metal toxicity to neurodevelopmental and neurodegenerative outcomes. His findings on co-exposure to heavy metals and stressors in rodent models echoed the idea that environmental toxicants can have chronic or long-lasting impacts on the well-being of the brain.

Emerging research topics, such as the influence of gut microbiota on neurodegeneration and gene-environment interactions in brain senescence, were presented by Dr Alexander Benedetto. These topics represent exciting frontiers in neuroscience, where interdisciplinary approaches are shedding light on previously underexplored mechanisms. The integration of microbiome research into the study of neurodegeneration provides new approaches for therapeutic interventions.

## Conclusion

The ISN Neuroscience School, Ondo 2024 successfully provided an in-depth investigation of gene-environment interactions in neurological disorders, combining lectures by faculties, students’ presentations, practical sessions and interactive forums. The different research presented emphasized the importance of using both genetic and environmental lenses to study neurodegeneration. The insights gained from this school, particularly in the context of model organisms and metal toxicity, will undoubtedly contribute to future research directions and therapeutic strategies in neuroscience.

## References

[BIO061998C1] Aryal, B. and Lee, Y. (2019). Disease model organism for Parkinson disease: Drosophila melanogaster. *BMB Rep.* 52, 250. 10.5483/BMBRep.2019.52.4.204 30545438 PMC6507844

[BIO061998C2] Bicca Obetine Baptista, F., Arantes, L. P., Machado, M. L., Da Silva, A. F., Marafiga Cordeiro, L., Da Silveira, T. L. and Soares, F. a. A. (2020). Diphenyl diselenide protects a Caenorhabditis elegans model for Huntington's disease by activation of the antioxidant pathway and a decrease in protein aggregation. *Metallomics* 12, 1142-1158. 10.1039/d0mt00074d 32453327

[BIO061998C3] Bilen, J. and Bonini, N. M. (2005). Drosophila as a model for human neurodegenerative disease. *Annu. Rev. Genet.* 39, 153-171. 10.1146/annurev.genet.39.110304.095804 16285856

[BIO061998C4] Bornhorst, J., Chakraborty, S., Meyer, S., Lohren, H., Große Brinkhaus, S., Knight, A. L., Caldwell, K. A., Caldwell, G. A., Karst, U., Schwerdtle, T. et al. (2014). The effects of pdr1, djr1. 1 and pink1 loss in manganese-induced toxicity and the role of α-synuclein in C. elegans. *Metallomics* 6, 476-490. 10.1039/C3MT00325F 24452053 PMC3954834

[BIO061998C5] Chen, P., Cheng, H., Zheng, F., Li, S., Bornhorst, J., Yang, B., Lee, K. H., Ke, T., Li, Y., Schwerdtle, T. et al. (2022). BTBD9 attenuates manganese-induced oxidative stress and neurotoxicity by regulating insulin growth factor signaling pathway. *Hum. Mol. Genet.* 31, 2207-2222. 10.1093/hmg/ddac025 35134179 PMC9262395

[BIO061998C6] Cordeiro, L. M., Machado, M. L., Da Silva, A. F., Baptista, F. B. O., Da Silveira, T. L., Soares, F. a. A. and Arantes, L. P. (2020). Rutin protects Huntington's disease through the insulin/IGF1 (IIS) signaling pathway and autophagy activity: study in Caenorhabditis elegans model. *Food Chem. Toxicol.* 141, 111323. 10.1016/j.fct.2020.111323 32278002

[BIO061998C7] Denzel, M. S., Lapierre, L. R. and Mack, H. I. D. (2019). Emerging topics in C. elegans aging research: Transcriptional regulation, stress response and epigenetics. *Mech. Ageing Dev.* 177, 4-21. 10.1016/j.mad.2018.08.001 30134144 PMC6696993

[BIO061998C8] Dunn, A. R., O'Connell, K. M. S. and Kaczorowski, C. C. (2019). Gene-by-environment interactions in Alzheimer's disease and Parkinson's disease. *Neurosci. Biobehav. Rev.* 103, 73-80. 10.1016/j.neubiorev.2019.06.018 31207254 PMC6700747

[BIO061998C9] Eid, A., Mhatre, I. and Richardson, J. R. (2019). Gene-environment interactions in Alzheimer's disease: a potential path to precision medicine. *Pharmacol. Ther.* 199, 173-187. 10.1016/j.pharmthera.2019.03.005 30877021 PMC6827882

[BIO061998C10] Gao, A. W., Uit De Bos, J., Sterken, M. G., Kammenga, J. E., Smith, R. L. and Houtkooper, R. H. (2018). Forward and reverse genetics approaches to uncover metabolic aging pathways in Caenorhabditis elegans. *Biochim. Biophys. Acta Mol. Basis Dis.* 1864, 2697-2706. 10.1016/j.bbadis.2017.09.006 28919364

[BIO061998C11] Gubert, P., Puntel, B., Lehmen, T., Fessel, J. P., Cheng, P., Bornhorst, J., Trindade, L. S., Avila, D. S., Aschner, M. and Soares, F. A. A. (2018). Metabolic effects of manganese in the nematode Caenorhabditis elegans through DAergic pathway and transcription factors activation. *Neurotoxicology* 67, 65-72. 10.1016/j.neuro.2018.04.008 29673961

[BIO061998C12] Gunter, T. E., Gerstner, B., Gunter, K. K., Malecki, J., Gelein, R., Valentine, W. M., Aschner, M. and Yule, D. I. (2013). Manganese transport via the transferrin mechanism. *Neurotoxicology* 34, 118-127. 10.1016/j.neuro.2012.10.018 23146871 PMC3576891

[BIO061998C13] Haroon, S., Li, A., Weinert, J. L., Fritsch, C., Ericson, N. G., Alexander-Floyd, J., Braeckman, B. P., Haynes, C. M., Bielas, J. H., Gidalevitz, T. et al. (2018). Multiple molecular mechanisms rescue mtDNA disease in C. elegans. *Cell Rep.* 22, 3115-3125. 10.1016/j.celrep.2018.02.099 29562168 PMC6106782

[BIO061998C14] Huang, W., Carbone, M. A., Lyman, R. F., Anholt, R. R. H. and Mackay, T. F. C. (2020). Genotype by environment interaction for gene expression in Drosophila melanogaster. *Nat. Commun.* 11, 5451. 10.1038/s41467-020-19131-y 33116142 PMC7595129

[BIO061998C15] Ijomone, O. M., Miah, M. R., Akingbade, G. T., Bucinca, H. and Aschner, M. (2020). Nickel-induced developmental neurotoxicity in C. elegans includes cholinergic, dopaminergic and GABAergic degeneration, altered behaviour, and increased SKN-1 activity. *Neurotox. Res.* 37, 1018-1028. 10.1007/s12640-020-00175-3 32034695 PMC13544494

[BIO061998C16] Ijomone, O. M., Weishaupt, A.-K., Michaelis, V., Ijomone, O. K. and Bornhorst, J. (2024). p38-and ERK-MAPK signalling modulate developmental neurotoxicity of nickel and vanadium in the Caenorhabditis elegans model. *Kinases Phosphatases* 2, 28-42. 10.3390/kinasesphosphatases2010003

[BIO061998C17] Machiela, E. and Southwell, A. L. (2020). Biological aging and the cellular pathogenesis of Huntington's disease. *J. Huntington's Dis.* 9, 115-128. 10.3233/JHD-200395 32417788 PMC7369111

[BIO061998C18] Migliore, L. and Coppedè, F. (2022). Gene–environment interactions in Alzheimer disease: the emerging role of epigenetics. *Nat. Rev. Neurol.* 18, 643-660. 10.1038/s41582-022-00714-w 36180553

[BIO061998C19] Okeowo, O. M., Anadu, V. E., Ijomone, O. K., Aschner, M. and Ijomone, O. M. (2024). Combined restraint stress and metal exposure paradigms in rats: unravelling behavioural and neurochemical perturbations. *Mol. Neurobiol.* 62, 4355-4376. 10.1007/s12035-024-04570-1 39443350 PMC12145149

[BIO061998C20] Pang, S. Y.-Y., Ho, P. W.-L., Liu, H.-F., Leung, C.-T., Li, L., Chang, E. E. S., Ramsden, D. B. and Ho, S.-L. (2019). The interplay of aging, genetics and environmental factors in the pathogenesis of Parkinson's disease. *Transl. Neurodegener.* 8, 1-11. 10.1186/s40035-019-0165-9 31428316 PMC6696688

[BIO061998C21] Soares, F. A., Fagundez, D. A. and Avila, D. S. (2017). Neurodegeneration induced by metals in Caenorhabditis elegans. In *Neurotoxicity of Metals. Advances in Neurobiology* (ed. M. Aschner and L. Costa) vol. 18, pp. 355-383. Springer, Cham. 10.1007/978-3-319-60189-2_18 28889277

[BIO061998C22] Wexler, N. S. (2004). Venezuelan kindreds reveal that genetic and environmental factors modulate Huntington's disease age of onset. *Proc. Natl Acad. Sci. USA* 101, 3498-3503. 10.1073/pnas.0308679101 14993615 PMC373491

